# Alkylpurine Glycosylase D Employs DNA Sculpting as a Strategy to Extrude and Excise Damaged Bases

**DOI:** 10.1371/journal.pcbi.1003704

**Published:** 2014-07-03

**Authors:** Bradley Kossmann, Ivaylo Ivanov

**Affiliations:** Department of Chemistry, Center for Diagnostics and Therapeutics, Georgia State University, Atlanta, Georgia, United States of America; University of Houston, United States of America

## Abstract

Alkylpurine glycosylase D (AlkD) exhibits a unique base excision strategy. Instead of interacting directly with the lesion, the enzyme engages the non-lesion DNA strand. AlkD induces flipping of the alkylated and opposing base accompanied by DNA stack compression. Since this strategy leaves the alkylated base solvent exposed, the means to achieve enzymatic cleavage had remained unclear. We determined a minimum energy path for flipping out a 3-methyl adenine by AlkD and computed a potential of mean force along this path to delineate the energetics of base extrusion. We show that AlkD acts as a scaffold to stabilize three distinct DNA conformations, including the final extruded state. These states are almost equivalent in free energy and separated by low barriers. Thus, AlkD acts by sculpting the global DNA conformation to achieve lesion expulsion from DNA. *N*-glycosidic bond scission is then facilitated by a backbone phosphate group proximal to the alkylated base.

## Author Summary

DNA repair efficiency is critically dependent on the function of DNA glycosylases. These versatile enzymes perform a remarkably discriminating search for DNA lesions, followed by damage-specific base extrusion into to the enzyme's active site and removal of the damaged bases. Our work elucidates the mechanism of *Bacillus cereus* AlkD, representative of a superfamily of alkylpurine glycosylase enzymes that function differently from all other known glycosylases. AlkD does not employ any direct contacts to the alkylated lesion. Instead, it relies on DNA backbone contacts to extrude the lesion's base-pairing partner. The alkylated base is flipped into solvent allowing N-glycosidic bond hydrolysis to occur with no apparent assistance from any protein side chains. Our work contributes to understanding of this unique base extrusion and excision strategy. We determined a minimum energy path for flipping out a 3-methyl adenine base by AlkD and computed an effective free energy profile for this transition. We show that lesion extrusion relies on DNA sculpting to break up the process into two steps characterized by low free energy barriers and a stable intermediate. AlkD provides a rigid scaffold to accommodate the three distinct DNA conformations and positions a phosphate group to facilitate scission of the alkylated base.

## Introduction

Despite its remarkable stability, DNA is subject to a variety of reactions. Left unchecked, these processes could impair the transmission of vital genetic information and threaten the integrity of the genome. To cope with genomic instability cells have evolved elaborate DNA repair mechanisms. Many cancer therapies are directly impacted by the efficiency of DNA repair. Antitumor drugs often act by inducing DNA lesions, thus, blocking replication in rapidly dividing cancer cells[1]. Upregulating DNA repair is a common mechanism in tumors to develop resistance to chemotherapy. Conversely, suppression of repair activity sensitizes cancer tissues to chemotherapies targeting DNA[1]–[4]. Specifically, alkylating agents can give rise to alkylpurine lesions[5] such as 3-methyl adenine (3 mA) and 7-methyl guanine (7 mG). Alkylpurine lesions carry a positive formal charge on the base, resulting in a sheared base pairing orientation and a comparatively labile *N-*glycosidic linkage that is prone to spontaneous hydrolysis. Not only are these lesions cytotoxic themselves, their propensity for spontaneous depurination could result in other, more deleterious forms of damage (*e.g.* single-strand or double-strand DNA breaks)[6], [7]. Alkylation lesions are processed by the cell's base excision repair (BER) machinery[8]–[10] to replace the damaged base with its correct Watson-Crick analog.

In BER, DNA N-glycosylases are the first line of defense against damage in genomic DNA. These enzymes efficiently and specifically recognize and excise single-base lesions[11], [12]. Structures of glycosylase enzymes reveal that DNA binding is accompanied by a multitude of conformational changes preceding active site chemistry. Specifically, nucleotide flipping (base extrusion) is a commonly employed strategy wherein a deoxynucleotide swings out of the DNA helix and is accommodated in the enzyme's catalytic pocket. This process has been the object of intense experimental focus for over two decades. Nonetheless, consensus has not been achieved regarding the pathways and molecular events that accompany base extrusion. Indeed, controversy has persisted regarding the precise role of the glycosylase (active or passive) in dislodging lesions from DNA. A number of alkylpurine-specific glycosylases have been characterized[13]–[16]. *Bacillus cereus* AlkD belongs to a unique superfamily of N3- and N7-alkylpurine glycosylases (present in all three domains of life) that function differently from all other known glycosylases[17]–[19]. Commonly, DNA glycosylases must flip damaged nucleotides out of the DNA base stack into damage specific pockets and must also accommodate the resulting DNA distortion by intercalating a side chain into the stack to replace the extrahelical nucleotide. To achieve efficient N-glycosidic bond scission, glycosylases must also provide side chains suitable to act as a general base in catalysis. By contrast, AlkD does not employ any direct contacts to the alkylated lesion. Instead, it relies on DNA backbone contacts to extrude the lesion's base-pairing partner. The alkylated base is flipped into the cytosol, allowing for hydrolysis to occur with no apparent assistance from any protein side chains[18].

## Results and Discussion

### Pathway and energetics of base pair opening and lesion extrusion by AlkD

**Figure 1:**
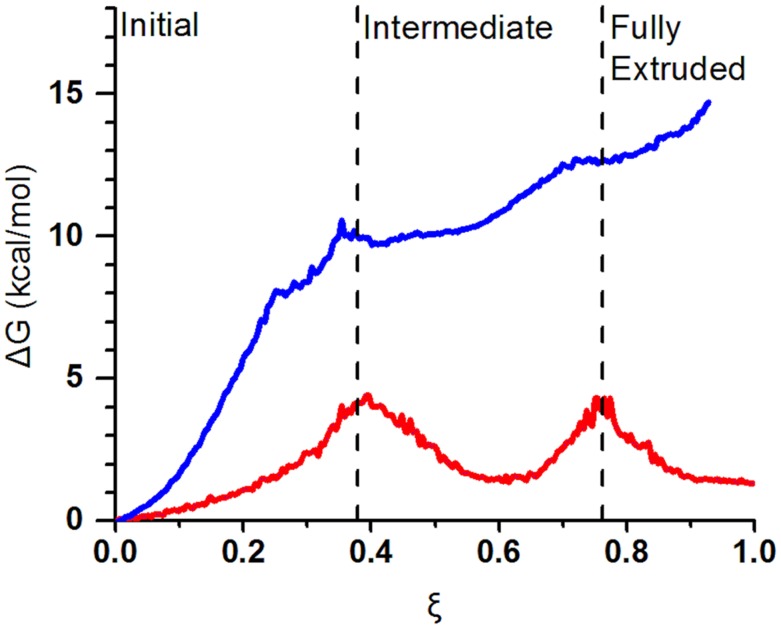
AlkD binding flattens the free energy landscape for lesion extrusion from DNA. Effective free energy profiles for base flipping in the presence (red line) and absence (blue line) of AlkD. The rmsd-based reaction coordinate, ξ was normalized to vary from 0 to 1.

Previous studies of DNA flipping[20]–[24] have relied primarily on intuitive reaction coordinates such as a pseudo torsional angle. However, AlkD works by sculpting the DNA backbone. A local reaction coordinate such as a pseudo dihedral is, in this case, inadequate. To describe such complex conformational transitions of DNA it is advantageous to employ path optimization methods such as the partial nudged elastic band (PNEB)[25], [26]. A key requirement is that the initial and final states in the transition be known. In this respect, crystal structures of AlkD with DNA containing a 3-deaza-3 mA or tetrahydrofuran (THF) are available to represent the pre-extrusion and post-excision complexes[18]. From these structures we constructed and equilibrated models for the initial and final AlkD/3 mA-DNA states and then computed a minimum energy path (MEP) for base extrusion of 3 mA by AlkD using PNEB (Figure S1). Umbrella sampling was then performed to describe the free energy profile of this conformational transition using molecular dynamics[27]. The reaction coordinate ξ for the transition was defined as ξ* =  rmsd_i_* – *rmsd_f_* where *rmsd_i(f)_* denotes root-mean-square deviation from the initial and final state. For clarity ξ was further normalized to vary from 0 to 1 (corresponding to the initial and final state, respectively). Among the advantages of ξ as a reaction coordinate is the ability to adequately describe global DNA bending induced by AlkD and to distinguish between concerted and sequential base flipping events. While the PNEB optimization involved all atoms of the AlkD/DNA complex, the definition of ξ involved rmsd over the nucleic acid heavy atoms alone. Additionally, we modeled B-form DNA with identical sequence and applied steered molecular dynamics to rotate the base opposite the 3 mA out of the base stack. Umbrella sampling was performed with the same protocol as for the AlkD/DNA complex, except ξ involved rmsd over the lesion pair and the base pairs immediately above or below in the stack. The obtained PMF profiles are shown in Figure 1. The reference profile suggests that base flipping in canonical B-DNA in the absence of AlkD proceeds with a steep initial rise in free energy as soon as the base departs from the stack. At ξ value of 0.4, ΔG reaches ∼10 kcal/mol and continues to increase to ∼14 kcal/mol albeit with a lesser slope afterward. The extruded state is thus represented by a broad plateau region with no apparent stabilization of the nucleotide anywhere outside the initial stacked conformation. Previous work on base flipping[20] in DNA is fully consistent with this view of the extrusion process.

**Figure 2:**
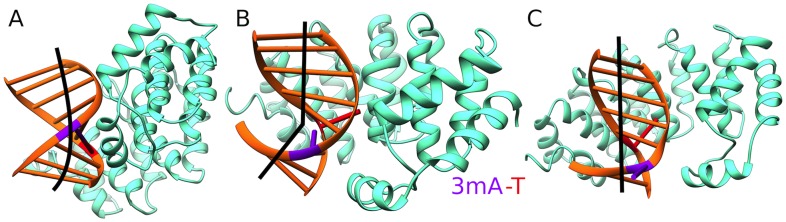
AlkD's sculpting of the DNA substrate results in three stable conformations along the flipping pathway. The three stable states in the AlkD/3 mA-DNA PMF. A) initial state; B) kinked intermediate state; and C) fully extruded (final) state. DNA bending is shown schematically in black.

Here we show that AlkD association to DNA substantially lowers the energy barrier for base flipping and provides a relatively flat free energy landscape characterized by three stable states (Figure 2) denoted as initial, intermediate and final (fully extruded). Notably, the barriers that separate these states are ∼3.9 and 3.0 kcal/mol, respectively. Two separate flipping events are resolved in the PNEB path and the PMF. The opposing base is extruded first and accommodated in a shallow pocket on the surface of AlkD. In this orientation the nucleotide is stabilized primarily through residue contacts to the DNA backbone, while the base itself remains solvent exposed. Two factors contribute to the observed barrier: (i) strain accompanying the opposing base rotation around the DNA backbone; and (ii) water penetration into the space previously occupied by the base in the DNA stack. The way AlkD relieves both of these factors after the initial barrier is to severely kink the DNA substrate while still preserving the 3 mA position in the base stack. Analysis of the umbrella sampling windows with the program Curves+[28] reveals that AlkD moderately bends the DNA in the initial state by 12.7°; severely kinks the DNA near the lesion in the intermediate state by 26.9°; and straightens the DNA to a negligible bend of 3.6° in the final state. The origin of the second barrier in the PMF is the rotation of the 3 mA lesion out of the DNA stack. Collapse of the water filled cavity left by the base and repositioning of two contacts to the lesion strand (Thr39 and Arg43) leads to the final fully extruded state. Base stack compression restores stacking interactions and removes the DNA kink. We note that the three stable states are almost equivalent in terms of free energy with initial and final differing by just ∼2 *k*_B_ *T* units of thermal energy. Thus, AlkD specifically stabilizes the extruded state allowing sufficient lifetime of 3 mA in the cytosol to accomplish hydrolysis. The low barriers among the three states could ensure frequent transitions on the ms timescale associated with base flipping.

### Structural determinants for DNA bending, double base flipping and catalysis by AlkD

**Figure 3:**
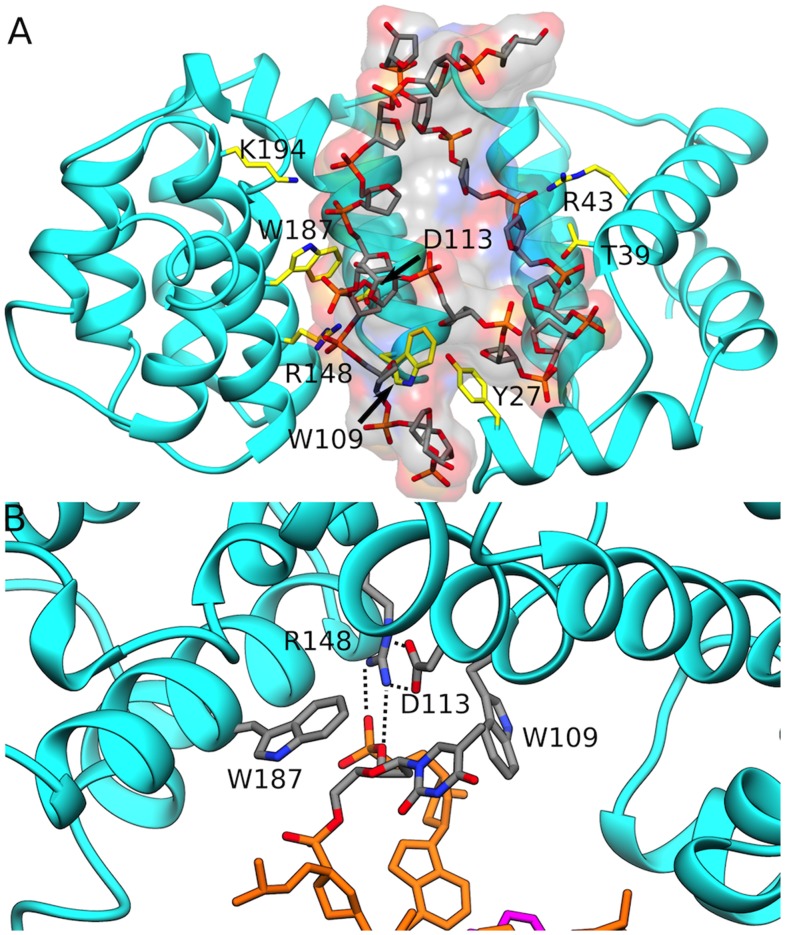
AlkD recognizes DNA through HEAT repeat motifs. A) Overall architecture of the AlkD-DNA complex with residues contacting the DNA backbone shown explicitly and labelled; B) The mode of recognition of the extrahelical thymine base opposite to the 3 mA lesion. DNA is shown in stick representation and as a transparent colored surface. AlkD is shown in cartoon representation.

As a complement to its unique strategy, AlkD is structurally comprised almost entirely of HEAT-repeat motifs, more commonly known to mediate protein-protein rather than protein-nucleic acid interactions[19]. In AlkD, repeats 2 through 6 are comprised of two antiparallel helices H1 and H2 that are oriented with a minor right-handed twist. The carboxy-terminal helix (H2) lines the concave surface of the DNA binding cleft and provides positively charged residues to recognize the non-lesion DNA strand (Figure 3a). The nucleotide opposite to the 3 mA is extruded into a shallow pocket on the protein surface with no specific contacts to the base (Figure 3b). The only major polar interaction involves the Arg148 residue, which doubly hydrogen bonds to the 3′ phosphate group of the extruded base. Two bulky tryptophan residues, W109 and W187, flank the DNA backbone to the 3′ and 5′ ends of the extruded base, sterically hindering rotations about the DNA backbone. These contacts are formed during the transition from the initial to the intermediate state and persist in the final state.

**Figure 4:**
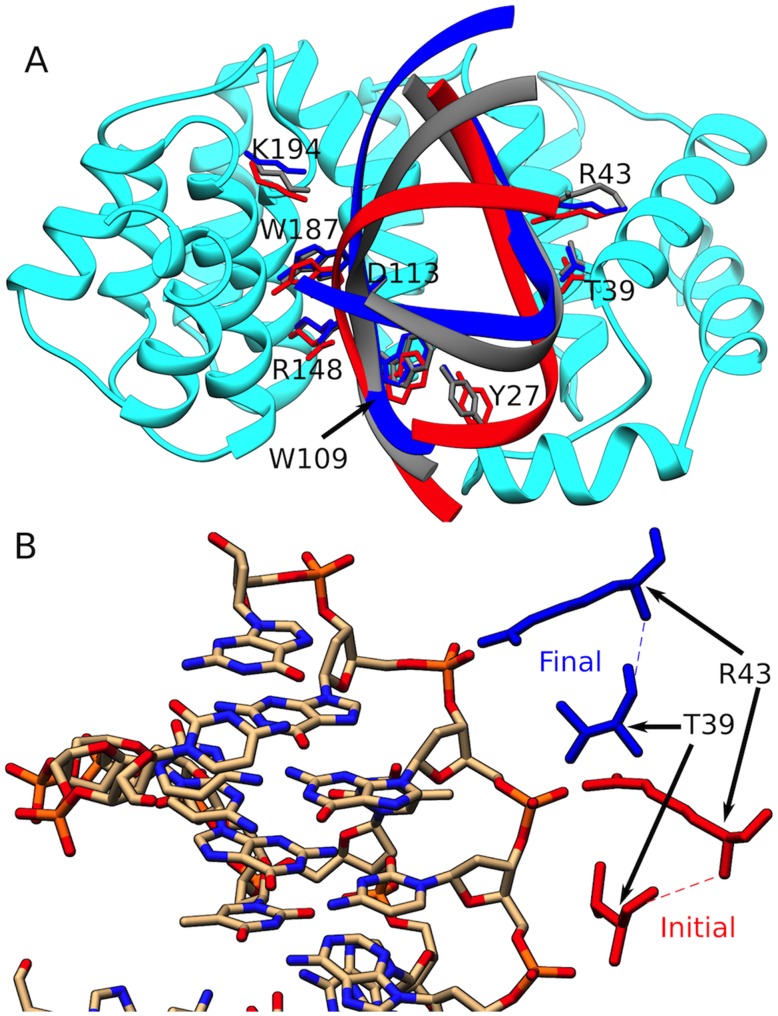
AlkD provides a scaffold to accommodate multiple DNA conformations with different degrees of bending. A) DNA substrate in the initial (red), intermediate (gray) and final (blue) states of AlkD base extrusion. AlkD is represented in cyan. Minimal side chain movement is sufficient to accommodate the DNA conformational transitions; B) Shift in hydrogen bonding contacts from the intermediate to the final state. Two AlkD residues, Arg43 and Thr39, making direct contacts to the lesion strand shift their binding positions along the DNA backbone.

Surprisingly, the large conformational transitions of the DNA are accompanied by only minor changes in the AlkD conformation (Figure 4). Energy decomposition with the NAMDenergy plugin of VMD[29] shows a rise in DNA conformational energy (primarily from the torsional component) and a concomitant increase in favorable protein-DNA interactions as base extrusion proceeds from initial to intermediate to final state (Figure S2). This corresponds to side chain adjustment of the residues contacting the DNA in these states (Figure 4a). At the same time we found that the change in rmsd from initial to final (computed over all heavy atoms of AlkD) was only ∼2 Å. Thus, AlkD requires no significant motion of the protein itself to flip and expose the 3 mA lesion. Instead, it provides a concave positively charged groove that is wide enough to accommodate multiple DNA conformations with different degrees of bending. Indeed, the only significant switch in AlkD-DNA contacts corresponding to the second barrier in the PMF was the observed repositioning of residues Thr39 and Arg43 with respect to the lesion strand (Figure 4b). These two residues shifted their hydrogen bonds by one phosphate group in the 3′ direction along the DNA backbone. Repositioning Thr39 and Arg43 has the dual effect of energetically stabilizing the final extruded 3 mA conformation and discouraging 3 mA reinsertion into the DNA stack.

**Figure 5:**
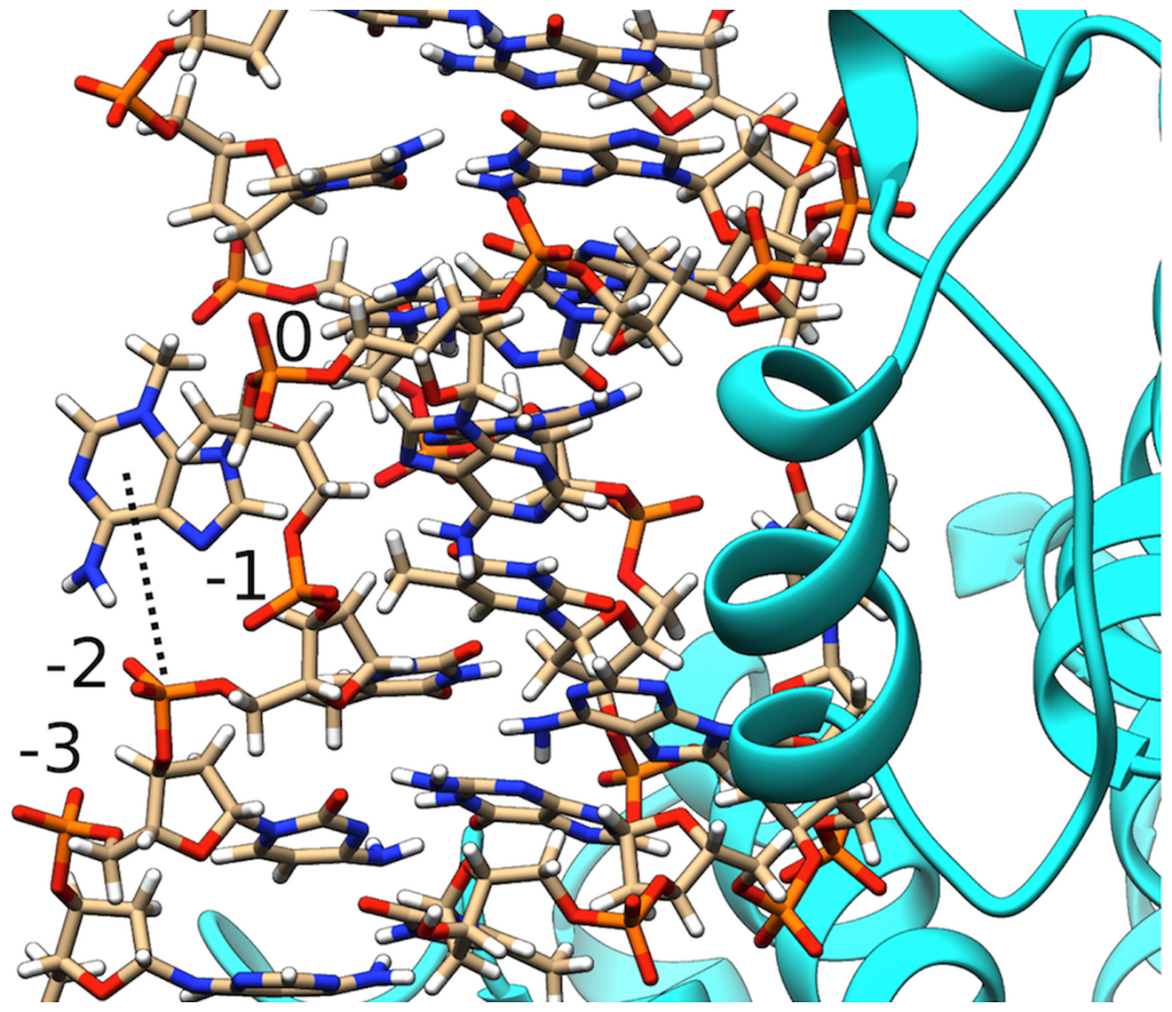
Persistent hydrogen bonding contact observed between the 3 mA base and the phosphate in position -2 along the lesion strand. Phosphate groups are numbered starting from the 3 mA lesion at position 0.

Sculpting the DNA substrate to promote 3 mA base eversion is obviously necessary for removal of the lesion. However, solvent exposure is not sufficient to explain the 230-fold catalytic rate enhancement (over the spontaneous rate of hydrolysis) offered by AlkD[19]. Recent biochemical evidence has pointed to AlkD's role in stabilizing a catalytically competent conformation by positioning a phosphate group in proximity to the lesion ribose. The mechanistic proposal is that the phosphate would serve a role analogous to a protein carboxylate group in stabilizing the developing positive charge on the lesion ribose in the transition state (TS)[30]. In our MD simulation of the extruded state we observe persistent direct interaction of the lesion with the phosphate in position -2 (Figure 5). However, the interaction occurs through hydrogen bonding to the 3 mA base rather than the ribose ring. This is reasonable as the 3 mA base carries a formal positive charge. Thus, it is possible AlkD employs an alternative strategy to stabilize the TS by differential hydrogen bonding to the 3 mA lesion. Altering hydrogen bonding to the base in the TS is not unprecedented and has also been proposed to contribute to catalysis by the prototypical glycosylase UDG[31]. This does not preclude a role for the phosphate stabilizing the ribose charge if the distance to the ribose decreases further in the TS complex.

### Conclusions

In summary, lesion extrusion by AlkD relies on DNA sculpting to break up the process into two steps, which are characterized by low free energy barriers and a stable intermediate. The end result is a flattened free energy landscape along the path from the initial to the fully extruded state. The rigid arrangement of HEAT-repeat helices results in a C-shaped, positively charged cleft providing a scaffold to accommodate three distinct DNA conformations with different degrees of bending. Finally, excision of the 3 mA base itself is dependent on the natural chemical instability of the alkylpurine *N*-glycosidic linkage and on a phosphate group suitably positioned to interact with the lesion in the extruded state. In this respect, the AlkD/DNA complex acts much like a DNAzyme, using the DNA backbone for catalysis. However, binding to AlkD's C-shaped cleft is required to achieve a catalytically competent conformation.

## Methods

### Model construction

Models for the pre- and post-extrusion states (denoted initial and final) were constructed from two AlkD/DNA crystal structures[18] (Protein Data Bank accession codes 3JX7 and 3JXZ, respectively). The partial nudged elastic band method (PNEB)[25], [26] requires an identical number of atoms in each replica of the band. Therefore, the DNA sequence in the final model was changed to match the DNA construct for the initial model. This construct comprised a 9 base-pair DNA duplex with lesion strand sequence 5′-ACT(3 mA)ACGGG-3′. The protein-DNA complexes were solvated with 9,977 TIP3P water molecules[32] in a box with dimensions 73.9×64.0×72.9 Å. Hydrogen atoms, Na^+^ counterions and solvent were introduced using the Xleap module of AMBER11[33] with the AMBER Parm99SB parameter set[34] and refined parameters for nucleic acids dihedrals (BSC0)[35]. 3 mA force field parameters were determined with the Antechamber module[36], [37] of AMBER. Partial charges for 3 mA were obtained by RESP fitting after DFT calculations performed at the BLYP/6-31G*[38] level with the Gaussian03[39] program.

### Equilibration protocol

The systems were equilibrated using the NAMD 2.8 code[27], [40] and minimized for 10,000 steps with harmonic restraints on the protein and nucleic acid atoms to remove unfavorable contacts. The systems were then gradually brought up to 300 K in the NVT ensemble while keeping the protein and nucleic acid atoms restrained. The equilibration was continued for another 2 ns in the NPT ensemble and the harmonic restraints were gradually released. Next, the simulations were continued for additional 11 ns of unrestrained molecular dynamics to ensure fully equilibrated initial and final states for PNEB.

### Path optimization

To determine a MEP connecting the pre- and post-extrusion AlkD configurations we employed the PNEB method - a chain-of-replicas method that involves concurrent optimization of a number of copies of the simulated system (denoted as replicas or beads). We chose to represent the path by a total of 30 replicas - 15 copies of the equilibrated initial and final states, respectively. By gradually spreading the replicas from the initial and final states we allow the PNEB optimization process to discover the MEP in a fully unbiased way. All atoms of the AlkD/DNA complex were included in the path optimization. Simulations were carried out with a 1-fs integration step in the NVT ensemble at 300 K. The minimum and maximum values for force constants between replicas were varied in from 0 to 4.5 kcal mol^−1^ Å^−2^ (k_min_) and from 0.25 to 4.5 kcal mol^−1^ Å^−2^ (k_max_). The PNEB protocol involved gradual ramping up of the force constant over 2 ns and subsequent scaling down to 2.0 kcal mol^−1^ Å^−2^ over another 4 ns. The PNEB band was optimized at 300K for 5ns and then gradually brought back to 0 K in the last 1 ns.

Additionally, we modeled canonical B-form DNA with sequence identical to the 9-mer from the AlkD/DNA complex. Equilibration involved 1,000 steps of minimization, 5 ps of NVT dynamics to bring the temperature to 300 K and 200 ps of dynamics in the NPT ensemble. After equilibration, we applied SMD to rotate the thymine base opposite the 3mA lesion out of the base stack. The base was rotated 180° through the minor groove with constant velocity for 4 ns.

### Umbrella sampling protocol

Umbrella sampling was performed to compute a PMF along the optimized PNEB path using the collective variables module of NAMD 2.8[27], [40] The collective variables module has a predefined RMSD variable (root-mean-square deviation of a group of atoms with respect to a reference structure). The module first calculates the best superposition of the atom group onto the set of reference coordinates before evaluating RMSD. The reaction coordinate (RC) was defined as ξ* =  rmsd_i_* – *rmsd_f_* where *rmsd_i(f)_* denoted root-mean-square deviation from the initial and final state, respectively. Each PNEB replica provided a configuration that was used to initiate an umbrella sampling window. Difference RMSD from initial and final was computed for this bead configuration and a harmonic umbrella potential (*k* = 3.0 kcal mol^−1^ Å^−2^) was applied and centered at the computed ξ value. The collective variable module internally distributes the applied force onto the selected atoms according to the definition of the RC to maintain small deviation from the center of each window. For the AlkD/DNA complex ξ was defined over the nucleic acid heavy atoms. For the reference DNA system, ξ was defined over the heavy atoms of the lesion pair and the base pairs immediately above or below in the DNA stack. The production runs were performed in the NPT ensemble (1 atm and 300 K) for 10 ns per window with the smooth particle mesh Ewald algorithm[41], short-range non-bonded cutoff at 10 Å and a switching function applied at 8.5 Å. The r-RESPA multiple timestep method[42] was employed with a 2-fs time step for bonded interactions, 2-fs for short-range non-bonded interactions and 4-fs for electrostatic interactions.

To analyze the results we used the weighted histogram analysis method (WHAM) as implemented in the code by Alan Grossfield[43] The first 2 ns from each window were considered equilibration and only the subsequent 8 ns were used for analysis. In total, 26 windows were sufficient to ensure uninterrupted coverage of the RC for WHAM calculations. Error bars were calculated by repeating the WHAM calculations in 2 ns increments (from 2 to 8 ns over the trajectories) and computing standard deviation (Figure S3). For the canonical B-form DNA control runs we carried out umbrella sampling with a 3.0 kcal mol^−1^ Å^−2^ harmonic restraint imposed on the RC with the same protocol as for the AlkD/DNA complex. Since the B-DNA systems required less time to reach convergence, we ran each window for 5 ns.

## Supporting Information

Figure S1 **Minimum energy path for base extrusion by AlkD.** The AlkD glycosylase is shown in green. The DNA configurations are taken from replicas along the PNEB path and colored from red (initial) to blue (final). The 3 mA lesion and its thymine partner are displayed as stick models with the same coloring scheme as the backbone.
(TIF)

Figure S2 **DNA conformational energy and DNA-protein interaction energy along the AlkD base extrusion pathway.** The values were computed by the NAMDenergy plugin in VMD. In computing the conformational energy (red points) only DNA atoms were considered; for the protein-DNA interaction energy (black points) only contributions from protein residues within 3.5 Å of DNA were included. Each point for the DNA conformational or DNA-protein interaction energy was obtained by averaging over a window from the umbrella sampling runs.
(TIF)

Figure S3 **Potential of mean force (PMF) for 3 mA base flipping by AlkD.** The rmsd-based reaction coordinate ξ was normalized to vary from 0 to 1. Error bars indicate standard deviation.
(TIF)
