# Supplementary figures and images for: Alkylpurine Glycosylase D Employs DNA Sculpting as a Strategy to Extrude and Excise Damaged Bases
Source: PLoS Comput Biol. 2014 Jul 3;10(7):e1003704. doi: 10.1371/journal.pcbi.1003704 (PMC4081403; doi:10.1371/journal.pcbi.1003704)

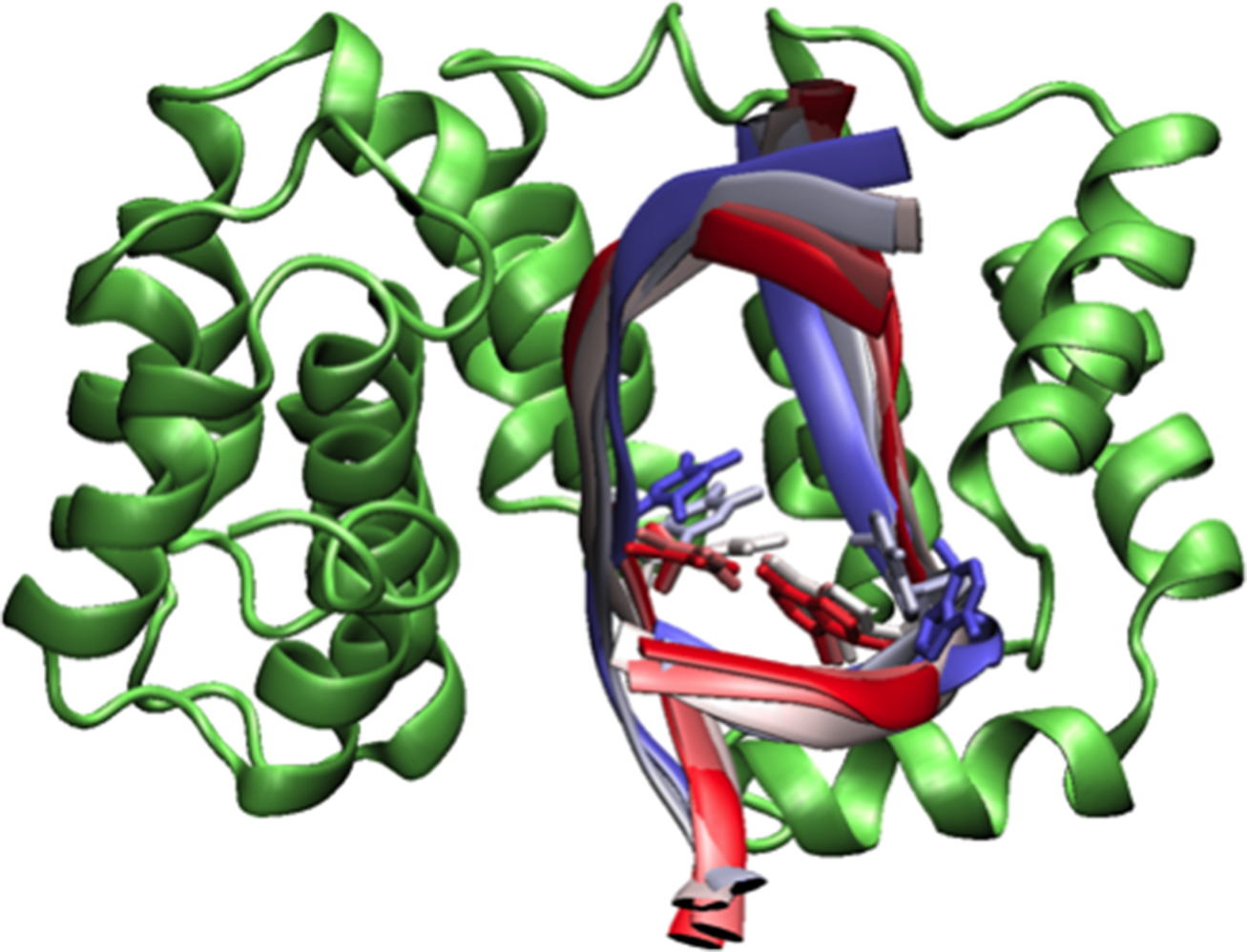

Supplement: Figure S1 — Minimum energy path for base extrusion by AlkD. The AlkD glycosylase is shown in green. The DNA configurations are taken from replicas along the PNEB path and colored from red (initial) to blue (final). The 3 mA lesion and its thymine partner are displayed as stick models with the same coloring scheme as the backbone. (TIF) [file pcbi.1003704.s001.tif]

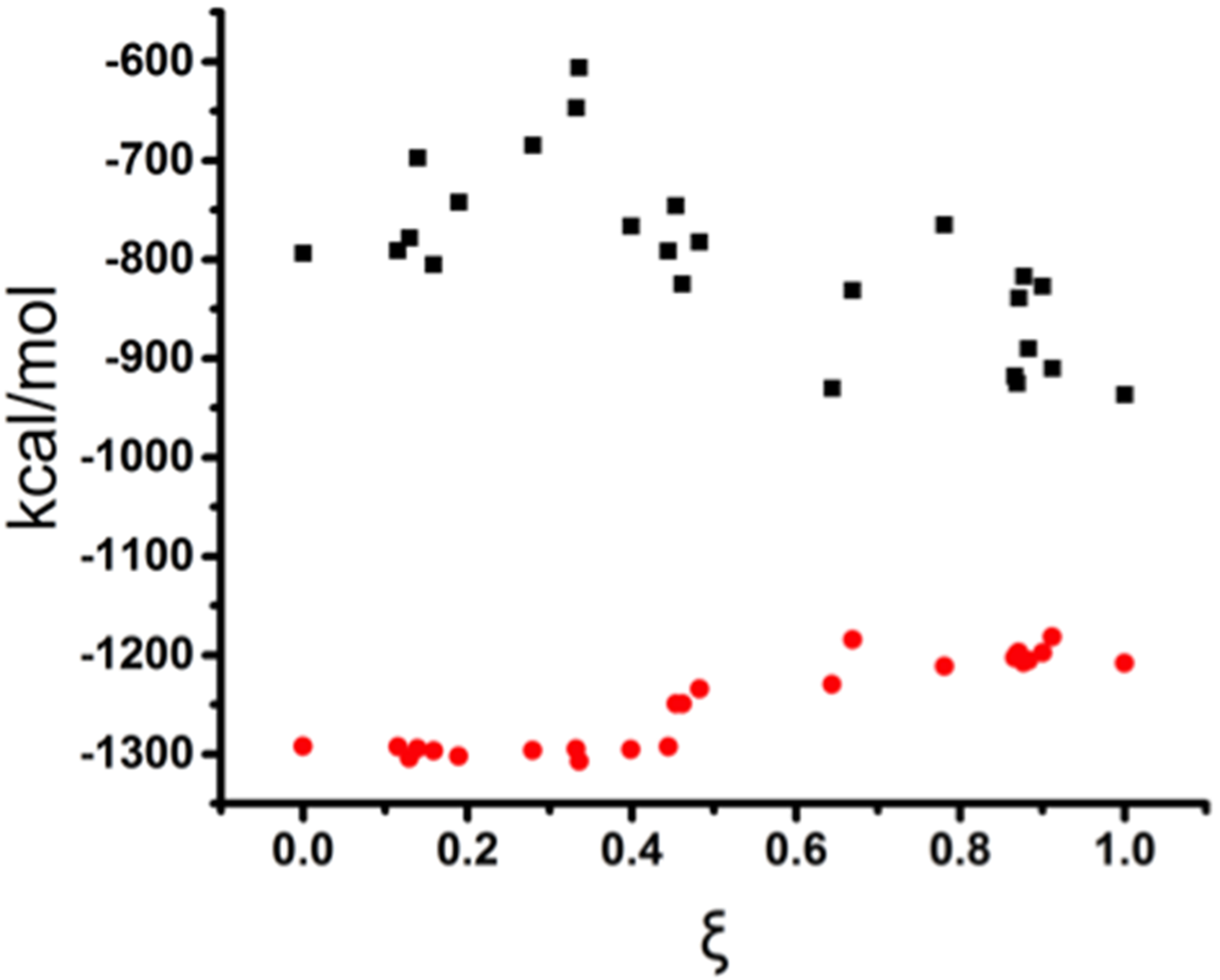

Supplement: Figure S2 — DNA conformational energy and DNA-protein interaction energy along the AlkD base extrusion pathway. The values were computed by the NAMDenergy plugin in VMD. In computing the conformational energy (red points) only DNA atoms were considered; for the protein-DNA interaction energy (black points) only contributions from protein residues within 3.5 Å of DNA were included. Each point for the DNA conformational or DNA-protein interaction energy was obtained by averaging over a window from the umbrella sampling runs. (TIF) [file pcbi.1003704.s002.tif]

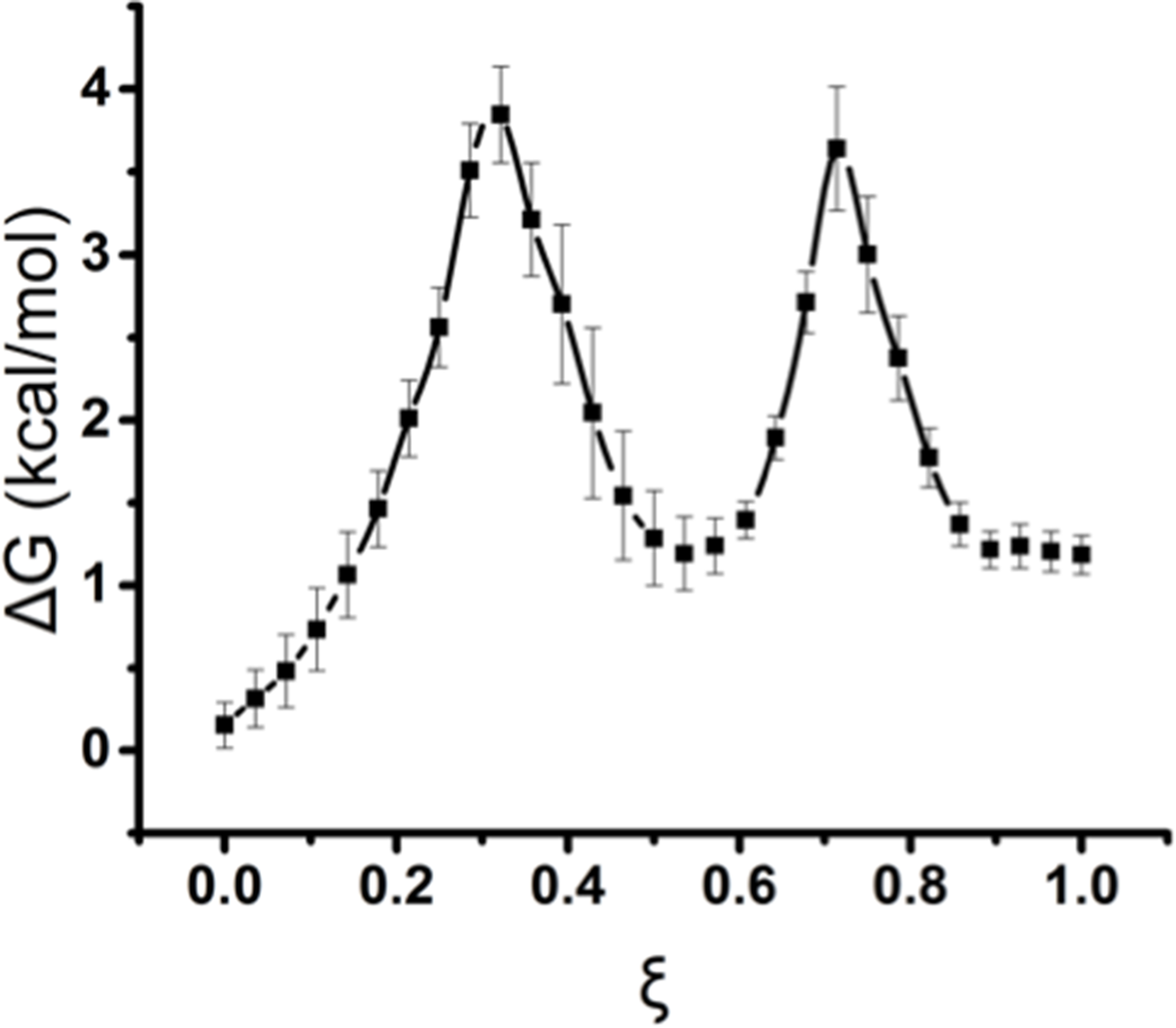

Supplement: Figure S3 — Potential of mean force (PMF) for 3 mA base flipping by AlkD. The rmsd-based reaction coordinate ξ was normalized to vary from 0 to 1. Error bars indicate standard deviation. (TIF) [file pcbi.1003704.s003.tif]
